# Monitoring Oxidative Status in Winemaking by Untargeted Linear Sweep Voltammetry

**DOI:** 10.3390/foods9060728

**Published:** 2020-06-03

**Authors:** Jelena Jeremic, Arianna Ricci, Gabriele Tacconi, Christine Lagarde-Pascal, Giuseppina Paola Parpinello, Andrea Versari

**Affiliations:** 1 Department of Agricultural and Food Sciences, University of Bologna, Piazza Goidanich 60, 47521 Cesena (FC), Italy; jelena.jeremic@ruffino.it (J.J.); arianna.ricci4@unibo.it (A.R.); andrea.versari@unibo.it (A.V.); 2 RUFFINO S.r.l.—A Constellation Brands Company-Winemaker, P.le Ruffino 1, 50065 Pontassieve (FI), Italy; Gabriele.Tacconi@ruffino.it; 3 Vinventions, Enology Team, 7 Avenue Yves Cazeaux, 30230 Rodilhan, France; christine.pascal@vinventions.com

**Keywords:** antioxidants, electrochemical technique, oxidation, winemaking

## Abstract

An electrochemical portable device based on linear sweep voltammetry was evaluated for studying the redox behavior of polyphenolic compounds in industrial scale winemaking to infer the effects of selected early processing steps on the vinification trials of Pinot gris, Chardonnay, Vermentino and Sangiovese grapes. For each sample, the redox behavior showed a distinctive voltammetric signal pattern related to the processing step during winemaking, therefore being useful as a potential fingerprint for wine identification and to provide insights about the phenolic content. For instance, there was a high correlation (*R*^2^ = 0.72) between the total phenolic compounds (PhenOx) and the easily oxidizable compounds (EasyOx), the latter representing approx. 30% on average of the total phenolics. Furthermore, the maceration of red grapes was concluded after 29 days based on information driven by the phenolics pattern. As expected, during alcoholic fermentation, white wines showed a lower content of phenolic compounds than those found in red wines, with an average ratio PhenOx/EasyOx of about 4.7, 5.0 and 3.6 for Chardonnay, Pinot gris and Vermentino, respectively. The portable tool with miniaturized disposable electrodes showed interesting analytical features that can be exploited for on-site and real-time quality control for monitoring change in phenolic composition during wine processing and storage, and for tailoring winemaking practices to enhance the color stability of products.

## 1. Introduction

Alcoholic fermentation is a key step in winemaking, during which many changes occur that affect the chemical composition and sensory properties of wine contributing to its complexity. In this view, there is a need for rapid and effective tools to monitor and drive the fermentation for exploiting the potential of each grape cultivar to avoid defects and to improve the characteristics of wine [1,2]. It is well known that the effect of oxygen on the chemical composition and sensory attributes of wine can be either positive or negative depending on several variables, including the timing and dosage of addition—either inadvertently or intentionally—along the winemaking process, from the initial harvesting of grapes to maturation in bottle [3,4,5]. In particular, oxidation is a major problem in winemaking as it can lead to development of color browning, bitterness of taste and a loss of varietal aroma and flavor of wine [6].

To protect wine from oxidation, sulfur dioxide is usually added during winemaking, however consumers’ concern about allergens in wine [7] has driven scientific research on the use of alternative winemaking protocols [8,9] and eventually the use of natural antioxidants, including oenological tannins [10].

In the last decade, grape and wine phenolic compounds have received rising interest from scientists and winemakers [11,12] due to their promising antioxidant activity, herein defined as any substance that delays, prevents or removes oxidative damage to a target molecule [13]. These compounds include tannins, flavan-3-ols, flavonols, phenolic acids, and stilbenes among others [14], whose antioxidant efficacy is highly dependent on the structural features of each molecule and is essentially related to the existence of hydroxyl function(s) in the aromatic moiety [15,16]. From the analytical perspective, there is a high correlation between total phenolic compounds and antioxidant capacity assayed as ABTS, DPPH and FRAP [17].

Despite recent advances in the understanding of wine oxidation [18,19,20], it is still problematic to monitor each winemaking step to predict the oxidative response of wine, which makes daily winemaking operations challenging and the final wine quality somewhat uncertain. With this in mind, there is a need for a rapid and reliable method to assess the wine oxidation alongside the entire winemaking process. Although several generic spectrophotometric methods for antioxidant testing are available (e.g., ABTS, DPPH, FOX, FRAP, TEAC, TRAP, etc.), the choice of method can have a great effect upon the results obtained due to the different chemistry of each antioxidant and sample tested [21,22,23], hence there is a need to find an ad-hoc antioxidant assay for grape and wine phenolics, bearing in mind their specific chemistry.

To this end, electrochemical methods also provide high potential for the investigation of antioxidant compounds, assessment of antioxidant capacity and measurement of electrochemical indexes by using different types of electrodes, including glassy carbon and modified gold electrodes [24]. In pioneer studies, Kilmartin et al. successfully applied cyclic voltammetry to characterize the antioxidant properties of wine antioxidants, including polyphenols, ascorbic acid, sulfur dioxide and glutathione [25,26,27,28]. Similarly, linear sweep voltammetry (LSV)—an electrochemical technique where the cell current is measured as a function of time and as a function of the potential between the indicator and reference electrodes—was recently used to study the mechanism of the electrochemical oxidation of rutin [29], and for the simultaneous determination of sulfite in fruit juice [30] and wine [31].

Although it has great potential as a scientific tool, the voltammetric technique has a major practical drawback due to the electrode fouling by wine phenolics, which is time consuming, due to the need for manual electrode cleaning procedures that can be avoided by using a new generation of disposable single-use electrodes. Recently, some studies have shown that voltammetry with disposable electrodes can be successfully used to characterize the oxidative patterns of white wines [32,33].

The aim of this study is to monitor for the first time the antioxidant pattern of red, rosé and white wine during early winemaking steps, at industrial scale, by using linear sweep voltammetry combined with a new disposable single-use electrode developed for phenolic compounds assay.

## 2. Materials and Methods

### 2.1. Experimental Design

Trials were designed to monitor the effects of selected winemaking steps (maceration, alcoholic fermentation and fining) on the chemical composition of red, rosé and white wines in terms of pooled phenolic compounds assessed by linear sweep voltammetry, combined with a disposable single-use electrode. Up to 116 measurements were done, 45 of which were during red grape vinification, and the remaining in white and rosè fining.

### 2.2. Samples

The wines—vintage 2019—used in the study were obtained at industrial level from different grape varieties, including Pinot gris, Chardonnay, Vermentino, and Sangiovese. All grapes were processed according to standard winemaking procedure directly at the winery (Ruffino, Pontassieve, Italy), and were analyzed on-site during the trials with the portable electrochemical device as described in the following sections.

### 2.3. Winemaking Practices

Standard winemaking practices in progress at Ruffino wineries were monitored on-site, including grape destemming and crushing (5 trials), fining (7 trials), maceration during red wine fermentation at 27 ± 2 °C (5 trials), white and rosé wine alcoholic fermentation at 17 ± 2 °C (20 trials) and red wine draining and pressing (10 trials). Each trial was monitored several times for a total of 116 samples. The experimental trials were run at production level, i.e., in a real winemaking company, therefore the selection of both grape cultivars and processing were driven by the actual needs at the winery.

### 2.4. Voltammetry

Electrochemical measurements were performed on-site using a portable device according to literature [32]. Briefly, a commercial Nomasense Polyscan P200 electrochemical analyzer (Vinventions, Schio, Italy) equipped with a disposable, miniaturized screen-printed triple-sensor—i.e., a three-electrode system including a single strip rectangular working electrode of 3.3 mm^2^, counter and reference electrodes—was used. For each analysis, one drop of a sample (ca. 50 μL) was poured on the sensor and the linear sweep voltammogram was recorded in the range 0–1200 mV at a scan rate of 100 mV/s under ambient conditions. Measurements were carried out without dilution of the sample, using a new sensor each time. All potentials are reported against the Ag/AgCl reference electrode.

Besides the LSV signal, the Polyscan is self-calibrated to provide four compositional indexes—all unitless—as follows: (i) EasyOx: compounds easily oxidized, including anthocyanins, thus quickly involved in the oxidation reactions; (ii) PhenOx: the set of all oxidizable compounds correlated with the index of Folin–Ciocalteu; (iii) IPT: Total Polyphenol Index commonly used by oenologists; and (iv) TAN/ACN ratio: for helping the winemaker to add the right amount of oxygen to wine during winemaking based on the tannin/anthocyanin ratio. The results of the Polyscan P200 calibration are available online [34].

### 2.5. Data Analysis

The data matrix consisted in 116 samples (red, rosé and white wines), 4 compositional indexes (EasyOx, PhenOx, IPT and TAN/ACN ratio), and the entire electrochemical signal up to 1200 mV with a scan rate every 10 mV for each sample (i.e., 120 data points). Data were analyzed with XLSTAT v. 2018.3 (Addinsoft, Paris, France) and The Unscrambler X v. 10.3 (Camo ASA, Oslo, Norway) using plots to visualize the effects of selected winemaking practices on the composition of wines. Linear discriminant analysis—commonly used when groups are known a priori—was carried out for modelling the difference between the classes of data to assess the adequacy of a classification of white wine during alcoholic fermentation, and disclosure of the most significant electrochemical signal using a stepwise selection of variables [35].

## 3. Results and Discussion

### 3.1. Linear Sweep Voltammetry

**Figure 1:**
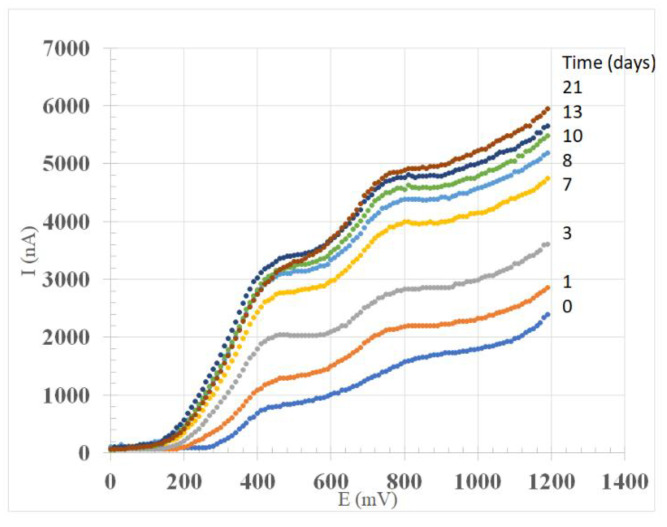
Linear sweep voltammetry during maceration of red wine Sangiovese up to 21 days.

Figure 1 shows an example of linear sweep voltammetry relating the electrode potential (E/mV) with the resulting current (l/nA), in which the signal was raised due to progressive oxidation of samples within the range 0–1200 mV—the high potential values used for oxidizing whole antioxidant compounds in red wine. The voltammograms showed a distinctive pattern with waves at approx. 440 and 750 mV. The former value matched the most easily oxidizable compounds present in red wines associated with catechol- and gallate-containing polyphenols [25], whereas the resulting current extents varied with increasing potentials due to different concentrations of whole phenolic compounds in the analyzed samples, including anthocyanins [36,37].

Moreover, the signals at day 13 and 21 showed an intersection at about 600 mV, with low values for the latter measurements (day 21) until 600 mV, where the signal increased to its upmost values at 1200 mV. It should be noted that simple phenolic compounds are oxidized at low values, whereas other phenolic compounds, with more difficultly oxidizable groups, generate peaks at higher potential values.

In red wines, flavan-3-ols along with oligomeric and polymeric tannins show the first voltammetric anodic peak at ~440 mV, which represents the oxidation of the catechol group on the B-ring, whereas the second oxidation peak at ~800–890 mV represents the oxidation of the resorcinol group on the A-ring. Moreover, the peak at about 680 mV is mainly associated with anthocyanins [25,38].

There was a high correlation (*R*^2^ = 0.72) between the entire scanned region (0–1200 mV) that estimates the total phenolic compounds (PhenOx) and the region corresponding to the low potentials (200–600 mV), which is related to more easily oxidizable compounds (EasyOx) such as catechins and caffeic, caftaric and gallic acids—the latter representing approx. 30% on average of the total phenolics.

In this trial, the maceration of Sangiovese red grapes was followed up to 21 days from grape destemming and crushing, during which the LSV signals showed an initial rapid rise (up to day 7) followed by a slowdown thus approaching a plateau at day 21 (Figure 1). The observed trend is consistent with the time course of phenolic extraction from red grapes, in which an almost instantaneous dissolution of ‘free’ solutes at the grape surface (i.e., leaching) is followed by diffusion of solutes from the grape interior [39]. Similarly, anthocyanin extraction during the maceration of the solid grape parts initially increases and reaches a maximum, however a subsequent decrease occurs due to degradation of anthocyanins and the condensation of tannins with anthocyanins [40]. 

### 3.2. Maceration Trials

**Figure 2:**
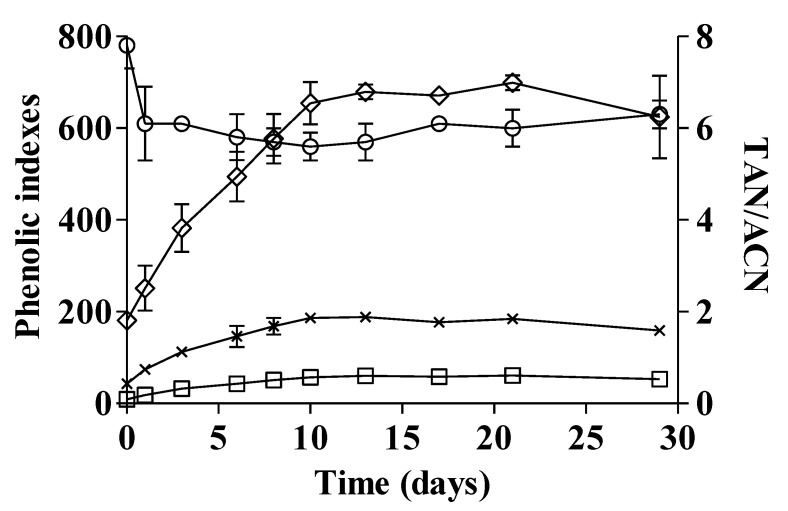
Time course of extraction of phenolic compounds during fermentative maceration of Sangiovese red grapes. Error bars below 5% CV are omitted to improve the quality of the plot. Legend: (□) IPT; (×) EasyOx, (◊) PhenOx; (○) TAN/ACN.

The trials on Sangiovese red grapes evaluated the change of selected phenolic indexes during the maceration of skins and seeds into juice during fermentation (Figure 2), which is a key process for tailoring red wine style through appropriate extraction of phenolics. Three electrochemical indexes related to phenolic composition—EasyOx, PhenOx and IPT—were monitored up to 29 days and, during this time, showed a specific pattern of extraction with the concentration increasing from the beginning of maceration until reaching a maximum plateau that can be fitted by an exponential approach to the final level. As expected, the easily oxidable phenolics (EasyOx) were a portion of the total oxidable phenolics (PhenOx), on average about 30%, whereas the TAN/ACN ratio levelled at around 6, which is typical for Sangiovese. These latter findings are consistent with the existence of a balance based on adsorption–desorption established between the anthocyanin content of the grape and the wine, and, when this balance has been reached, no further anthocyanins can be extracted from grape skins into the wine [41]. From this point, a downward trend occurs, mainly due to oxidation, precipitation, modifications in their structure and adsorption in yeast cell walls. Similarly, tannin extraction is a diffusion process with different extraction kinetics between skin and seed tannins; the former are readily solubilized together with anthocyanins, whereas the latter show a slow diffusion rate, which is faster when alcohol aids dissolution of the seed cuticle [6].

### 3.3. Alcoholic Fermentation Trails

During the white wine alcoholic fermentation, the technique was tested on three white grapes—Pinot gris (*n*. 14), Chardonnay (*n*. 10) and Vermentino (*n*. 4)—and discriminant analysis was applied to the electrochemical signals for classifying samples into groups and to better understand the relationships that may exist among the variables.

**Figure 3:**
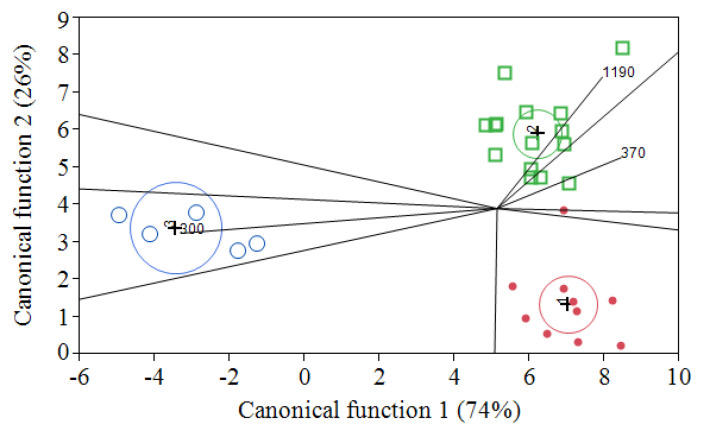
Linear discriminant analysis of Pinot gris (□), Chardonnay (*λ*) and Vermentino (○) grapes during alcoholic fermentation at 17 °C.

Discriminant analysis—applied on a selected range of the electrochemical signal, i.e., from 300 to 1200 mV, to reduce the background noise—successfully grouped the 28 white wines according to their cultivar (Figure 3). Stepwise procedure was applied for the selection of variables, the first ten of which (300, 370, 670, 680, 770, 780, 940, 970, 1000 and 1190 mV) allowed a satisfactory result with only one Chardonnay sample misclassified. It is noteworthy that most of the selected signals were related to a high oxidation voltage corresponding to chemical compounds less easily oxidized, which seems the main driver for disclosing the specific qualitative pattern among grape cultivars. The approach herein presented seems suitable to monitor the effects of wine oxidation as well. Indeed, the use of voltammogram fingerprinting as a successful tool for monitoring white wine oxidation status was originally proposed using cyclic voltammetry (from 200 to 1200 mV at a scan rate of 100 mV/s) in combination with supervised multivariate analysis [42].

**Figure 4:**
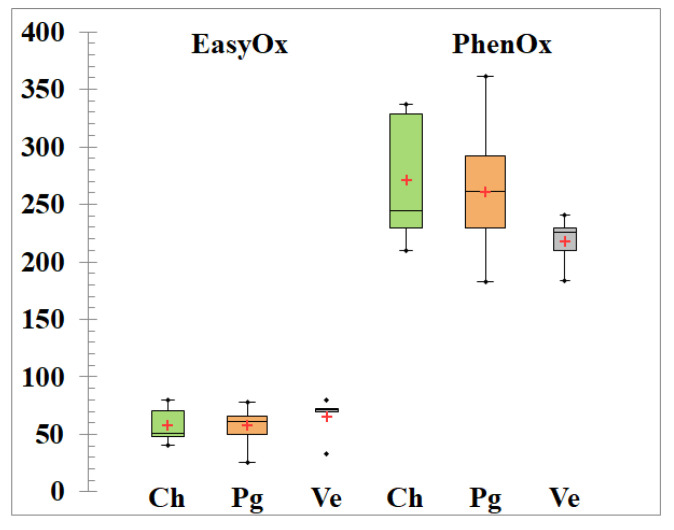
Box plot of EsyOx and PhenOx indexes for Chardonnay (Ch), Pinot gris (Pg) and Vermentino (Ve) grapes during alcoholic fermentation at 17 °C.

As expected, the content of phenolic compounds found in white wines during alcoholic fermentation (Figure 4) was lower than those found in Sangiovese red wines (Figure 2), with an average ratio PhenOx/EasyOx of about 4.7, 5.0 and 3.6 for Chardonnay, Pinot gris and Vermentino, respectively. This finding stimulates further links with literature trying to gain insight into the astringent attributes of wine. Indeed, the skin contact process increases the levels of phenolics in the final wines, and the effect of prefermentative maceration on wine composition depends on the contact time [43] and temperature [44]. In particular, a comparative study on the vinification of Vermentino grapes found the following total polyphenolic compounds: 82, 112, 141, 162 and 522 mg/L, for hyperoxygenation, control, reductive conditions, prefermentative cryomaceration (5 °C × 24 h) and extended maceration, respectively [45]. Similar studies on Chardonnay grapes confirmed that below 6 °C the increase in total polyphenolic compounds in wine is negligible and peaked at ca. 440 mg/L [46].

### 3.4. Fining Trials

**Figure 5:**
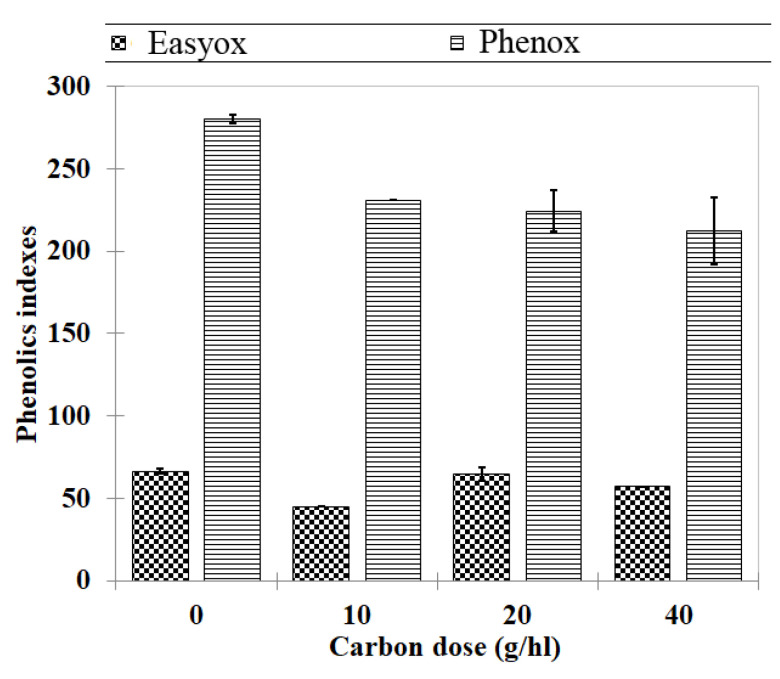
Effect of fining with carbon on the composition of Pinot gris must.

Early fining on Pinot gris musts with activated carbon from charcoal was used, rather than later in wine, to remove the oxidizable phenolics before any effect on color is caused, including pinking, and to improve the color of rosè wines as well. The total oxidable polyphenolics (PhenOx) showed significant decreases following the use of activated charcoal up to 40 g/hl, whereas the effect on EasyOx seemed negligible (Figure 5). Commercial carbons have different degrees of uptake of anthocyanins from white wines prepared from red grapes and excess tannins from wine [47], and these differences in removing polyphenolics depend on the type of wine variety and the method of preparation of the activated carbon [48]. In our trials, the small effect on EasyOx index, which accounts for anthocyanins as well, is probably related to the very low concentration of these pigments in the Pinot gris samples, making it difficult to selectively adsorb them onto carbon for obtaining appreciable removal. It is well known that Pinot gris wine is at risk of pinking—a term that refers to the salmon-red blush color that may appear in white wines produced exclusively from white grape varieties—of which the threshold of anthocyanins needed for the pink color visualization in wine is 0.3 mg/L [49].

Concerning bioactive compounds, it is well known that the antioxidant activity of wines is positively correlated (up to *r* = 0.94) with total phenolic contents [17,50]. The correlation between total phenolics measured spectrophotometrically and the signal measured during voltammetric analysis is positive (up to *R*^2^ = 0.99) as well [51,52,53], including for the polyscan device herein used (*R*^2^ = 0.91) [54], in which the sum of each oxidation current per potential increment is defined as the ‘antioxidant power’ of the wine or grape must, which is well correlated (*R*^2^ = 0.98) with 420 nm AU values [55].

## 4. Conclusions

The availability of appropriate tools and procedures is essential for process monitoring in cellars to ensure high quality products. In this view, a simple and rapid approach based on disposable screen-printed voltammetric sensors was effectively applied for on-site monitoring of the phenolic profile of white and red grapes during the early steps of the winemaking process at the cellar. The compositional data gathered represents a valuable aid for improving process management, particularly for skin maceration and must fining practices, enabling oenologists to take timely corrective action.

For example, grape musts with low EasyOx and/or PhenOx values would most likely profit from pressing under inert conditions, whereas grape musts with increasing values would probably need mild and strong fining treatments, respectively, and eventually the compositional values would drive the decision of hyperoxygenation of must or microxygenation of wines according to the strategy selected. Furthermore, controlling the extraction of phenolic compounds during maceration of red grapes helps to define the extent of the process, including the timing of saignée for rosé wines and racking for reds, in order to improve the quality of wines. In conclusion, monitoring raw materials, in-process samples, and final wines would allow for the building of quality control charts for improving whole-product quality management.
